# Three Different Biopesticides Against *Megalurothrips usitatus* (Thysanoptera: Thripidae) and Their Toxicological and Biochemical Impacts

**DOI:** 10.3390/biology14111619

**Published:** 2025-11-18

**Authors:** Zuying Fu, Ziyu Cao, Changyu Xiong, Yifan Cui, Yuanrun Cheng, Ying Wang, Rong Zhang, Chang Liu, Wei Sun, Liping Ban, Yao Tan, Shuhua Wei

**Affiliations:** 1 Institute of Plant Protection, Ningxia Academy of Agricultural and Forestry Sciences, Yinchuan 750002, China; 19896202015@163.com (Z.F.); czy200218@163.com (Z.C.); 15950550587@163.com (C.X.); sy20233243683@cau.edu.cn (Y.C.); cyr20010216@163.com (Y.C.); wangying108@163.com (Y.W.); yczhrnx@163.com (R.Z.); liuchangamy@126.com (C.L.); swlymyy@163.com (W.S.); 2 College of Horticulture and Plant Protection, Inner Mongolia Agricultural University, Hohhot 010010, China; 850310.tanhuaf4@163.com; 3 College of Biological Science & Engineering, North Minzu University, Yinchuan 750002, China; 4 College of Grassland Science and Technology, China Agricultural University, Beijing 100193, China; lipingban@cau.edu.cn

**Keywords:** biopesticides, biocontrol bacteria, biological control, botanical pesticide, entomopathogenic fungus, *Megalurothrips usitatus* (Bagrall)

## Abstract

The laboratory toxicity and physiological effects of three biopesticides—the entomopathogenic fungus *Beauveria bassiana*, the biocontrol bacterium *Bacillus safensis*, and the botanical insecticide *Artemisia ordosica* essential oil—were evaluated against *Megalurothrips usitatus*, a major pest of alfalfa. All three biopesticides caused concentration-dependent mortality in *M. usitatus.* Among them, *B. bassiana* showed the highest corrected mortality (88.42%) and the shortest median lethal time (LT_50_ = 4.51 days), indicating strong insecticidal activity. The median lethal concentration (LC_50_) values at 7 days post-treatment were 4.48 × 10^5^ CFU/mL for *B. bassiana*, 1.67 × 10^5^ CFU/mL for *B. safensis*, and 2.907 mg/L for *A. ordosica* essential oil. Moreover, exposure to high concentrations of each agent significantly altered the activities of the protective enzyme peroxidase (POD) and the detoxifying enzyme glutathione S-transferase (GST). Our study supports the use of these biopesticides for thrips management and provides a scientific basis for the eco-friendly and sustainable control of alfalfa pests.

## Simple Summary

The bean thrips, *Megalurothrips usitatus*, is a major pest of alfalfa. Owing to the issues of pesticide resistance and environmental harm associated with conventional chemicals, we evaluated three eco-friendly biopesticides for their control potential. Laboratory bioassays demonstrated that the entomopathogenic fungus *Beauveria bassiana*, the bacterium *Bacillus safensis*, and the essential oil from *Artemisia ordosica* were all effective against *M. usitatus*. Among them, *B. bassiana* exhibited the fastest action and highest mortality. Furthermore, these biopesticides induced sublethal physiological effects by modulating the activity of key enzymes involved in detoxification and stress response. Our findings confirm the potential of these natural agents as sustainable alternatives for integrated thrips management.

## 1. Introduction

*Megalurothrips usitatus* (Bagrall) (Thysanoptera: Thripidae) is a significant pest of legume crops, particularly in the alfalfa fields of Ningxia [1,2,3]. However, overreliance on chemical pesticides for its control has led to the development of pest resistance, ecological imbalance, and environmental contamination [4,5,6]. Consequently, biological control—the use of natural enemies and pathogenic microorganisms—has emerged as a sustainable and promising component of integrated pest management strategies [7].

Entomopathogenic fungi constitute a cornerstone of microbial biopesticides. They typically infect hosts via cuticular penetration: spores adhere to the insect cuticle, germinate, and secrete a suite of cuticle-degrading enzymes to breach the host integument, ultimately leading to host death through nutrient depletion and toxin production [8,9]. Among them, *Beauveria bassiana* is a well-characterized species with demonstrated efficacy against various thrips, including *Thrips palmi*, *Frankliniella occidentalis*, and *Thrips tabaci* [10,11]. Its practical potential is underscored by field reports, such as a control efficacy exceeding 74% against thrips in greenhouse peppers following the application of *B. bassiana* wettable powder [12].

*Bacillus* species are another major group of biocontrol microorganisms, valued for their diverse insecticidal mechanisms and favorable environmental safety profiles, while *Bacillus safensis* is a Gram-positive, spore-forming bacterium [13]. Studies have shown that feeding *B. safensis* L4-1 to housefly pupae delayed development and growth [14], and its fermentation broth exhibited significant mosquito-repellent activity [15]. Moreover, *B. safensis* can markedly reduce the pupation, eclosion, and survival rates of *Ostrinia furnacalis*, underscoring its potential as a biocontrol agent [16].

Botanical insecticides derived from plant extracts and essential oils show significant potential. Species from the Solanaceae, Stemonaceae, Fabaceae, and Asteraceae families are particularly promising, as their rich content of bioactive compounds like sesquiterpene lactones and flavonoids can disrupt insect physiology and behavior [17,18]. *Artemisia ordosica*, a semi-shrub of the genus *Artemisia* (Asteraceae family), is a dominant species widely distributed in desert areas of northern and northwestern China [19]. Yu et al. [20] demonstrated that *A. ordosica* exhibits broad-spectrum antibacterial activity, providing a material foundation for developing plant-derived pesticides.

To elucidate the sublethal physiological responses of *M. usitatus* to biopesticide exposure, we focused on two key enzymes: peroxidase (POD) and glutathione S-transferase (GST). POD is a crucial component of the insect antioxidant system, mitigating oxidative stress by scavenging free radicals [21,22,23]. Conversely, GST is a central detoxification enzyme that conjugates glutathione to a broad spectrum of xenobiotics, including insecticides and plant allelochemicals, and its induction is a common response to pesticide stress [24,25,26,27,28]. Therefore, the activities of POD and GST serve as sensitive biomarkers for assessing insect metabolic adaptation and defense mechanisms [29], providing insights into the mode of action of the tested biopesticides.

Given the economic importance of *M. usitatus* in alfalfa production and the need for sustainable control solutions, this study aimed to evaluate the efficacy of three distinct biopesticides: the entomopathogenic fungus *Beauveria bassiana*, the biocontrol bacterium *Bacillus safensis*, and the botanical insecticide derived from *Artemisia ordosica* essential oil. We assessed their laboratory toxicity against *M. usitatus* and, concurrently, investigated their sublethal effects on the activities of POD and GST to unravel associated physiological disruptions. This integrated bioassay–biochemistry approach provides a comprehensive basis for selecting effective biocontrol agents and contributes to the development of environmentally sustainable IPM strategies for alfalfa.

## 2. Materials and Methods

### 2.1. Insects for Testing

*Megalurothrips usitatus* were collected from an alfalfa planting base in Xixia District, Yinchuan City, Ningxia Hui Autonomous Region (38°38′59″ N, 106°9′6″ E). The collected thrips were reared in an artificial climatic chamber maintained at 25 ± 1 °C and 14 L:10 D photoperiod, using cowpeas (*Vigna unguiculata*) as host plants. Adults of *M. usitatus* were subsequently selected for experiments.

### 2.2. Formulation of Different Concentrations

The entomopathogenic fungi formulations *Beauveria bassiana* IPPM34315 wettable powder (15 billion spores/g) and biocontrol bacteria *Bacillus safensis* spore suspension (1 × 10^7^ CFU/mL) were provided by the College of Grass Science and Technology, China Agricultural University.

Conidial suspensions of *Beauveria bassiana* and spore suspensions of *Bacillus safensis* were prepared in sterile water containing 0.05% (*v*/*v*) Tween-80. The initial concentration of each suspension was determined using hemocytometer [30]. The number of spores per milliliter was calculated based on the average count per large square (1 mm × 1 mm), multiplied by a factor of 1 × 10^4^ [31]. Subsequently, the B. bassiana suspension was serially diluted to obtain final concentrations ranging from 1 × 10^4^ to 1 × 10^8^ CFU/mL. Similarly, the *B. safensis* suspension was adjusted to concentrations ranging from 1 × 10^4^ to 1 × 10^7^ CFU/mL for use in subsequent bioassays. *Artemisia ordosica* essential oil was used as a botanical pesticide. It was initially emulsified in Tween-80 (1:1, *v*/*v*) and subsequently diluted with Tween-80 to prepare test solutions at concentrations of 1.5625, 3.125, 6.25, 12.5, and 25 mg/L. Using Tween-80 to treat *M. usitatus* as a blank control. The emulsifier concentration was kept below 1% (*v*/*v*) in all treatments and the control to negate its own insecticidal effects.

### 2.3. Determination of Laboratory Virulence of Biopesticides Against M. usitatus

The toxicity of various agents against *M. usitatus* was assessed using a leaf-dipping bioassay [32]. Fresh cowpea pods were washed, rinsed thoroughly to remove any pesticide residues, and air-dried. Subsequently, they were cut into uniform segments (2 cm in length) and immersed for 15 s in one of the following: suspensions of *B. bassiana* or *B. safensis*, aqueous solutions of *Artemisia ordosica* essential oil, or an emulsifier control (Tween-80). After air-drying, the treated segments were transferred into Petri dishes. Each dish was infested with 25 adult thrips, and each concentration was replicated three times. All dishes were maintained under controlled conditions: 25 °C, 60 ± 5% relative humidity, and a 14 h:10 h light:dark photoperiod.

### 2.4. Effects of Different Agents Treatments on Enzyme Activity of M. usitatus

To evaluate the effects of high-concentration exposures, adult *M. usitatus* were treated with the following agents at designated high concentrations: *Beauveria bassiana* at 1 × 10^9^ CFU/mL, *Bacillus safensis* at 1 × 10^7^ CFU/mL, and an aqueous emulsion of *Artemisia ordosica* essential oil at 25 mg/L. The treatment followed the leaf-dipping protocol described in Section 2.3. For each agent, four independent biological replicates (*n* = 3) were established in a randomized experimental design. Each replicate, consisting of 25 insects confined in a Petri dish, was considered an experimental unit. Using Tween-80 to treat *M. usitatus* as a blank control.

Enzyme activities for POD and GST were quantified using specific commercial assay kits (Solarbio, Beijing, China), following the manufacturer’s protocols. The assay principles are based on monitoring absorbance changes at specific wavelengths. Briefly, frozen *M. usitatus* samples were homogenized in ice-cold extraction buffers provided with the kits. The homogenates were centrifuged at 10,000× *g* for 10 min at 4 °C in centrifuge (Fresco17, Thermo Fisher Scientific, Waltham, MA, USA), and the resulting supernatants were collected as crude enzyme extracts. The POD activity was determined by tracking the oxidation of guaiacol at 470 nm. The GST activity was measured by monitoring the conjugation of glutathione (GSH) with 1-chloro-2,4-dinitrobenzene (CDNB) at 340 nm. All absorbance readings were recorded using a microplate reader. Concurrently, the total protein concentration of each extract was determined using the Coomassie Brilliant Blue G-250 method (Bradford method) by measuring the absorbance at 595 nm, with bovine serum albumin (BSA) as the standard [33]. The specific enzyme activities were ultimately calculated and normalized to the total protein content, expressed as units per milligram of protein (U/mg prot).

### 2.5. Data Analysis

Corrected mortality rates were calculated using Abbott’s formula [34]. Corrected Mortality (%) = [(Mortality in treatment − Mortality in control)/(1 − Mortality in control)] × 100. The toxicity regression equations, median lethal times (LT_50_), median lethal concentrations (LC_50_), and their corresponding 95% confidence intervals were determined using probit analysis in IBM SPSS Statistics 26.0 (IBM Corp, Armonk, NY, USA).

The corrected mortality data were analyzed using a generalized linear model (GLM) with a binomial distribution and a logit link function to assess the effects of treatment concentration and time. For the enzyme activity data, the assumptions of normality and homogeneity of variances were verified. Since these assumptions were met, the data were subjected to one-way analysis of variance (ANOVA).

In all analyses, Duncan’s new multiple range test was used for post hoc multiple comparisons to distinguish significant differences among treatment means. A significance threshold of *p* < 0.05 was applied for all statistical tests. All figures were generated using GraphPad Prism 9.5.0 (GraphPad Software, San Diego, CA, USA).

## 3. Results

### 3.1. Determination of Laboratory Toxicity of Biopesticides Against M. usitatus

#### 3.1.1. Corrected Mortality and Lethal Time (LT_50_) of *B. bassiana* on *M. usitatus*

**Figure 1:**
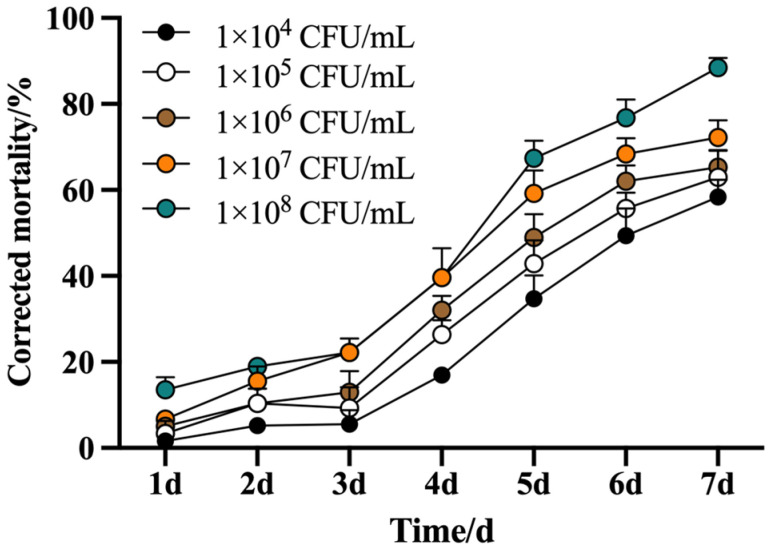
Corrected mortality of *M. usitatus* under different concentration of *B. bassiana.* The standard error of the means (three replicates) is indicated by the error bars. Bars showing different letters were significantly different from one another on different days after treatment.

The corrected mortality of *M. usitatus* exhibited a significant positive correlation with the concentration of *B. bassiana* suspensions (Figure 1). A clear dose and time-dependent response was observed. After a 5 d, the mortality rates at the two highest concentrations (1 × 10^7^ and 1 × 10^8^ CFU/mL) surpassed 50%, reaching 59.18% and 67.34%, respectively. By day 7, the efficacy increased further, with mortality rates rising to 76.79% and 88.42% at the same concentrations. The corresponding median lethal times (LT_50_) for these concentrations were calculated to be 4.87 d and 4.51 d, respectively (Table 1), indicating a faster lethal effect at the higher concentration.

#### 3.1.2. Corrected Mortality and Lethal Time (LT_50_) of *B. safensis* on *M. usitatus*

**Figure 2:**
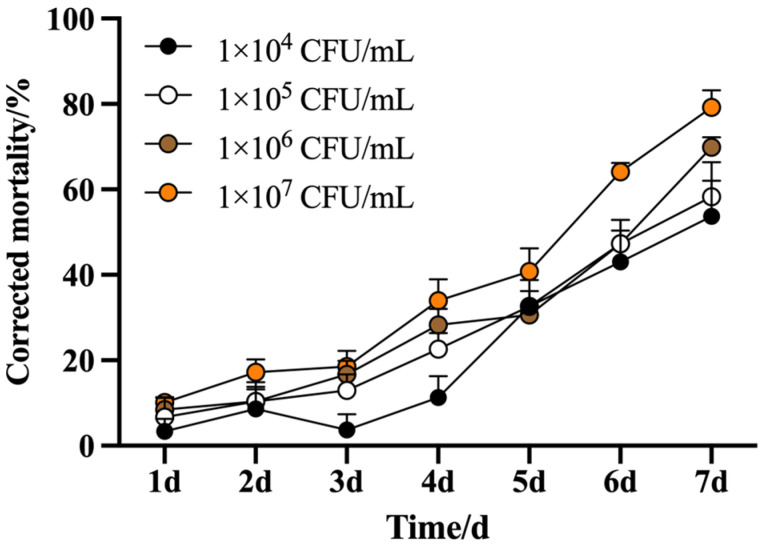
Corrected mortality of *M. usitatus* under different concentration of *B*. *safensis.* The standard error of the means (three replicates) is indicated by the error bars. Bars showing different letters were significantly different from one another on different days after treatment.

The pathogenicity of *B. safensis* against *M. usitatus* was showed to be both concentration and time dependent (Figure 2). Mortality increased in a robust concentration dependent manner, with the highest concentration (1 × 10^7^ CFU/mL) achieving a corrected mortality of 55.69% after 6 d, exceeding the 50% threshold. After 7 d, the mortality rates at concentrations of 1 × 10^6^ and 1 × 10^7^ CFU/mL increased significantly to 64.13% and 79.16%, respectively. The corresponding median lethal time (LT_50_) values for these concentrations were determined to be 5.76 d and 5.08 d (Table 2), indicating a more rapid lethal effect at the higher bacterial concentration.

#### 3.1.3. Corrected Mortality and Lethal Time (LT_50_) of *A. ordosica* essential oil on *M. usitatus*

**Figure 3:**
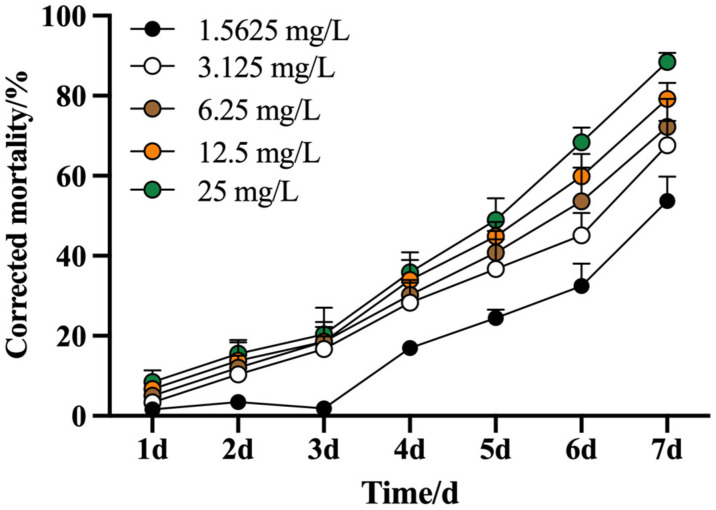
Corrected mortality of *M. usitatus* under different concentration of *A. ordosica.* The standard error of the means (three replicates) is indicated by the error bars. Bars showing different letters were significantly different from one another on different days after treatment.

The insecticidal efficacy of *A. ordosica* essential oil against *M. usitatus* exhibited a significant concentration- and time-dependent relationship (Figure 3). A critical efficacy threshold was surpassed after 5 days of exposure at 6.25 mg/L, with a corrected mortality rate of 53.58%. The lethal effect was substantially enhanced by day 7, where mortality rates reached 68.35% and 88.42% at concentrations of 12.5 and 25 mg/L, respectively. The corresponding median lethal times (LT_50_) for these concentrations were calculated to be 5.18 d and 4.91 d (Table 3), demonstrating a faster action at the higher application rate.

#### 3.1.4. Laboratory Toxicity of Different Agents Against *M. usitatus*

The corrected mortality and lethal time assays demonstrated that the virulence of all three biopesticides—*B. bassiana*, *B. safensis*, and *A. ordosica* essential oil increased with prolonged exposure. Therefore, we calculated the laboratory toxicity of the three agents after 7 d. LC_50_ of *B. bassiana* treatment was 4.48 × 10^5^ CFU/mL against *M. usitatus*, and LC_90_ was 4.08 × 10^8^ CFU/mL. LC_50_ of *B. safensis* treatment was 1.67 × 10^5^ CFU/mL against *M. usitatus* and LC_90_ was 1.84 × 10^8^ CFU/mL. LC_50_ of *A. ordosica* essential oils treatment against *M. usitatus* was 2.907 mg/L and LC_90_ was 22.134 mg/L (Table 4).

### 3.2. Protective Enzyme Peroxidase (POD) and Detoxifying Enzyme Glutathione S-Transferase (GST) Activity of M. usitatus

#### 3.2.1. POD Enzyme Activity of *M. usitatus*

**Figure 4:**
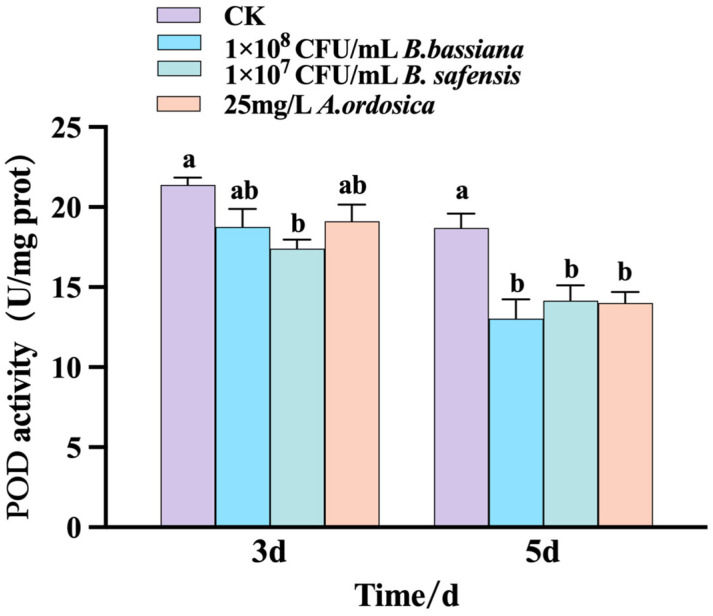
Effects of different treatments on Peroxidase activity of *M. usitatus.* The standard error of the means (three replicates) is indicated by the error bars. Bars showing different letters were significantly different from one another on different days after treatment.

Changes in POD enzyme activity in *M. usitatus* were measured 3 and 5 d after treatment with 1 × 10^8^ CFU/mL *B. bassiana*, 1 × 10^7^ CFU/mL *B. safensis*, and 25 mg/L *A. ordosica* essential oil. Results showed significantly lower POD activities in all treatment groups compared to the control (*p* < 0.05). The lowest POD activity occurred at 3 d in *M. usitatus* treated with 1 × 10^7^ CFU/mL *B. safensis*. By 5 d, all treatments exhibited further reductions in POD activity relative to the control, with the lowest activity observed in *B. bassiana* treated (1 × 10^8^ CFU/mL) insects (Figure 4).

#### 3.2.2. GST Enzyme Activity of *M. usitatus*

**Figure 5:**
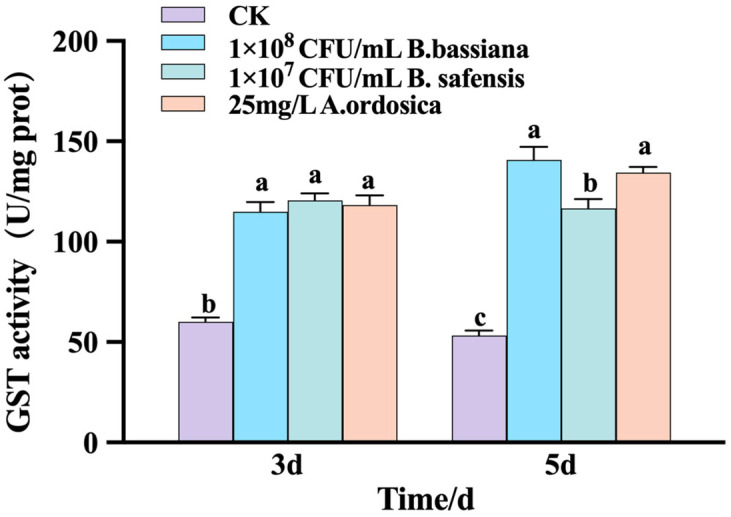
Effects of different treatments on the Glutathione-S transferase activity of *M. usitatus.* The standard error of the means (three replicates) is indicated by the error bars. Bars showing different letters were significantly different from one another on different days after treatment.

Changes in GST enzyme activity in *M. usitatus* were measured 3 and 5 d after treatment with 1 × 10^8^ CFU/mL *B. bassiana*, 1 × 10^7^ CFU/mL *B. safensis*, and 25 mg/L *A. ordosica* essential oil. Results showed significantly higher GST activity in all treatment groups compared to the control (*p* < 0.05). At 3 d, no significant differences in GST activity were observed among the three treatments. By 5 d, GST activity in control group decreased, while all agent-treated groups exhibited increased activity. Notably, GST activity in *B. safensis* treated insects (1 × 10^7^ CFU/mL) was significantly lower than in those treated with *B. bassiana* and *A. ordosica* essential oil (*p* < 0.05) (Figure 5).

## 4. Discussion

The escalating problems of pest resistance and adverse environmental impacts associated with synthetic pesticides highlight an urgent need for sustainable control alternatives. Our study demonstrates that the entomopathogenic fungus *Beauveria bassiana*, the bacterium *Bacillus safensis*, and *Artemisia ordosica* essential oil all possess significant insecticidal activity against *M. usitatus*, confirming their potential as eco-friendly biopesticides.

Treatment with *B. bassiana* at 1 × 10^7^ and 1 × 10^8^ CFU/mL and *A. ordosica* essential oil at 6.25 mg/L resulted in mortality rates exceeding 50% within 5 days. The highest mortality (88.42%) was observed after 7 days of exposure to *B. bassiana* at 1 × 10^8^ CFU/mL, with a corresponding LC_50_ value of 4.48 × 10^5^ CFU/mL. The LT_50_ values for *B. bassiana* (at 1 × 10^7^ and 1 × 10^8^ CFU/mL) and *A. ordosica* essential oil (at 25 mg/L) were all below 5 d, with the shortest LT_50_ (4.51 d) recorded for *B. bassiana* at 1 × 10^8^ CFU/mL—consistent with previous findings by Yang et al. [35]. This supports the potential of *B. bassiana* in thrips management, possibly through the secretion of specific proteases or virulence factors that compromise host immunity [36]. *B. safensis* also demonstrated considerable efficacy, causing 79.16% mortality at 1 × 10^7^ CFU/mL after 7 days, with LT_50_ and LC_50_ values of 5.08 d and 1.67 × 10^5^ CFU/mL, respectively. Despite these promising results, research on *B. safensis* as a bioinsecticide remains limited [37,38], warranting further mechanistic and field-level studies. *A. ordosica* essential oil showed strong insecticidal activity against *M. usitatus*, in line with earlier reports of its efficacy against *Mythimna separata* [39]. At 25 mg/L, the LT_50_ was 4.91 d, likely resulting from multiple modes of action including contact, repellent, fumigant, and growth-inhibitory effects. Moreover, botanical pesticides such as *A. ordosica* oil are characterized by high environmental compatibility and full biodegradability, positioning them as ecologically safe alternatives to conventional pesticides [40,41].

The sublethal physiological effects of these biopesticides were evident in the significant alterations of key enzyme activities. The consistent suppression of peroxidase (POD) across all treatments indicates a compromised antioxidant defense system, potentially rendering the thrips more vulnerable to oxidative stress induced by the biopesticides [42]. Conversely, the significant induction of glutathione S-transferase (GST) suggests an active detoxification response [43]. This elevated GST activity not only underscores its role in the metabolic response to these natural compounds but also raises the possibility of cross-tolerance, highlighting a potential risk for the development of resistance that should be monitored [44].

While highly promising, the translation of these laboratory results to the field must address challenges such as environmental stability and consistent performance under varying agro-climatic conditions. A strategic approach to overcome these hurdles lies in formulating synergistic combinations, for instance, pairing the rapid action of *A. ordosica* oil with the persistent infectivity of *B. bassiana*. Such combinations could enhance overall efficacy, lower required doses, and mitigate resistance development. Furthermore, while this study provides insights through POD and GST activities, a more comprehensive mechanistic understanding awaits the examination of a broader spectrum of physiological responses, including other detoxification enzymes and digestive enzyme profiles.

We recommend scaling up regional demonstrations that integrate microbial and botanical formulations. Concurrent ecological benefit assessments from plot to landscape levels should be conducted to unlock the full potential of integrated thrips management. Such advances will strengthen the practical impact of biopesticides and contribute to sustainable, green strategies in pest control.

## 5. Conclusions

In conclusion, this study demonstrates that the entomopathogenic fungus *Beauveria bassiana*, the biocontrol bacterium *Bacillus safensis*, and *Artemisia ordosica* essential oil all possess significant insecticidal activity against *Megalurothrips usitatus*, establishing their potential as sustainable alternatives to chemical pesticides. Our results reveal distinct virulence profiles among the following agents: *B. bassiana* proved to be the fastest-acting with the highest overall mortality, whereas *B. safensis* exhibited the highest intrinsic potency with the lowest LC_50_ value, and *A. ordosica* essential oil displayed a balanced efficacy through presumed multiple modes of action. Furthermore, exposure to these biopesticides induced significant sublethal physiological disruptions in *M. usitatus*, characterized by a consistent suppression of the protective enzyme POD and a concurrent induction of the detoxifying enzyme GST. These enzymatic responses not only provide insights into the modes of action but also highlight the potential for metabolic resistance development. The complementary strengths of these biopesticides suggest that future research should focus on developing synergistic combinations to enhance efficacy, overcome field application challenges, and delay resistance, thereby advancing the goals of green and sustainable integrated pest management in alfalfa cultivation.

## Figures and Tables

**Table 1:** LT_50_ of *M. usitatus* under different concentration of *B. bassiana.*

Treatment	Concentration (CFU/mL)	Toxicity Regression Equation	LT_50_/d	95% Confidence Interval	X^2^	Correlation Coefficient	*p*
*B. bassiana*	1 × 10^4^	y = 3.07x − 5.54	6.09	5.63~6.72	17.30	0.89	<0.01
	1 × 10^5^	y = 2.42x − 4.2	5.66	5.15~6.26	13.98	0.91	<0.01
	1 × 10^6^	y = 2.44x − 4	5.12	4.71~5.65	12.60	0.93	<0.01
	1 × 10^7^	y = 2.07x − 3.27	4.87	4.42~5.30	10.46	0.91	<0.01
	1 × 10^8^	y = 2.59x − 3.85	4.51	4.11~4.84	5.54	0.91	<0.01

**Table 2:** LT_50_ of *M. usitatus* under different concentration of *B. safensis.*

Treatment	Concentration (CFU/mL)	Toxicity Regression Equation	LT_50_/d	95% Confidence Interval	X^2^	Correlation Coefficient	*p*
*B. safensis*	1 × 10^4^	y = 1.91x − 3.66	6.72	5.88~9.41	11.33	0.88	=0.01
	1 × 10^5^	y = 2.29x − 4.17	6.13	5.62~6.89	12.13	0.87	<0.01
	1 × 10^6^	y = 2.23x − 3.9	5.76	5.21~6.40	9.20	0.90	<0.01
	1 × 10^7^	y = 2.05x − 3.29	5.08	4.52~5.56	5.99	0.91	<0.01

**Table 3:** LT_50_ of *M. usitatus* under different concentration of *A. ordosica.*

Treatment	Concentration (mg/L)	Toxicity Regression Equation	LT_50_/d	95% Confidence Interval	X^2^	Correlation Coefficient	*p*
*A. ordosica*	1.5625	y = 2.76x − 5.24	6.62	6.12~7.51	23.63	0.89	<0.01
	3.125	y = 2.16x − 3.82	5.85	5.35~6.49	17.19	0.83	<0.01
	6.25	y = 2.15x − 3.67	5.57	5.07~6.13	13.14	0.86	<0.01
	12.5	y = 2.09x − 3.41	5.18	4.71~5.63	7.84	0.88	<0.01
	25	y = 2.37x − 3.67	4.91	4.50~5.26	12.00	0.87	<0.01

**Table 4:** Toxicity of different agents against *M. usitatus* under laboratory conditions.

Agents	Time/d	Toxicity Regression Equation	LC_50_	LC_90_	X^2^	Correlation Coefficient	*F*	*p*
*B. bassiana* (CFU/mL)	7	y = 0.27x − 0.08	4.48 × 10^5^	4.08 × 10^8^	2.17	0.84	10	0.01
*B. safensis* (CFU/mL)	7	y = 0.31x − 0.02	1.67 × 10^5^	1.84 × 10^8^	2.27	0.79	8	0.03
*A. ordosica* (mg/L)	7	y = 0.06x − 0.18	2.91	22.13	4.17	0.75	12	0.03

## Data Availability

The data presented in this study are available on request from the corresponding author.
